# Green Synthesis of Silver Nanoparticles from *Aloe vera*: Antibacterial Potential Against Cyanobacteria from an Andean Lagoon

**DOI:** 10.3390/life16071132

**Published:** 2026-07-07

**Authors:** Arnold Solano, Antonio Vega, José Davalos-Monteiro, Daniel Cabrera-Valle, Carlos Loyo-Dávila, Lenin Ramírez-Cando, Fernando Villalba-Meneses, Diego Almeida-Galárraga, Vladimir Bonilla, Maria Baldeon-Calisto, Raúl Dávalos Monteiro, Patricia Acosta-Vargas

**Affiliations:** 1 School of Biological Sciences and Engineering, Yachay Tech University, Urcuqui 100119, Ecuador or asolano@usm.cl (A.S.); antonio.vega@yachaytech.edu.ec (A.V.); lramirez@yachaytech.edu.ec (L.R.-C.); gvillalba@yachaytech.edu.ec (F.V.-M.); dalmeida@yachaytech.edu.ec (D.A.-G.); rdavalos@yachaytech.edu.ec (R.D.M.); 2 Departamento de Ingeniería Química y Ambiental, Universidad Técnica Federico Santa María, Avenida España 1680, Valparaiso 2390123, Chile; 3 Sinopec Tech Middle East LLC, Dhahran 34465, Saudi Arabia; jose.stme@sinopec.com; 4 Facultad de Ciencia e Ingeniería en Alimentos y Biotecnología, Universidad Técnica de Ambato, Av. Los Chasquis y Río Payamino, Ambato 180207, Ecuador; da.cabrera@uta.edu.ec; 5 School of Chemical Sciences and Engineering, Yachay Tech University, Hda. San José s/n y Proyecto Yachay, Urcuqui 100119, Ecuador; cloyo@yachaytech.edu.ec; 6 Facultad de Ingenierías Digitales y Tecnologías Emergentes, Universidad Internacional del Ecuador, Quito 1701518, Ecuador; febonillave@uide.edu.ec; 7 Porter B. Byrum School of Business, Wingate University, Wingate, NC 28174, USA; m.baldeoncalisto@wingate.edu; 8 Departamento de Ingeniería Industrial, Universidad San Francisco de Quito—USFQ, Quito 170901, Ecuador; 9 Intelligent and Interactive Systems Laboratory, Universidad de Las Américas, Quito 170125, Ecuador

**Keywords:** silver nanoparticles, *Aloe vera*, cyanobacteria, bacterial inhibition, green synthesis

## Abstract

This work describes an efficient and environmentally friendly method for the synthesis of silver-based nanostructures through a green route using *Aloe vera* extract as a reducing agent, silver nitrate (AgNO_3_) as a precursor, and polyvinylpyrrolidone (PVP, 10 kDa molecular weight) as a stabilizing agent. The formation of these structures was supported by UV–Vis spectroscopy, where a surface plasmon resonance (SPR) band was observed between 425 and 460 nm. Scanning electron microscopy revealed predominantly spherical features in the 300–500 nm range; however, the distinction between primary nanoparticles and aggregates cannot be conclusively established from SEM alone. EDX analysis indicated a silver content of 59.96 wt%. Antibacterial assays performed in Z8 medium demonstrated a reduction in cyanobacterial growth with increasing dosage, with complete inhibition observed at ≥20 μL (nominal MIC = 1.77 mg mL^−1^, based on precursor estimation). Total dissolved solids and absorbance measurements exhibited a decreasing trend with increasing concentration (effect size = 0.87, p<0.001), supporting an inhibitory effect under the tested conditions. These findings suggest potential antibacterial activity. However, this study should be considered exploratory, and further work is required to elucidate the underlying mechanisms.

## 1. Introduction

Nanoparticles have attracted special attention in recent decades due to their extensive applications in life sciences, including biomedical sciences [1,2,3,4], biotechnology and environmental engineering [5,6,7,8,9], and fluorescence and magnetic imaging [10,11]. Moreover, nanotechnology has advanced several fields, including catalysis, sensing, electronics, and photonics [12,13]. Some examples of nanomaterials with potential applications in medicine, biomedicine, and electronics include quantum dots, carbon nanotubes, biosensors, nanoengineered computer chips, and nanofibers [14,15,16,17].

Among the different types of nanoparticles, AgNPs have been extensively studied due to their remarkable physical and chemical properties, such as high electrical conductivity, thermal stability, distinctive optical characteristics, and strong antibacterial activity [18,19,20,21]. These properties make AgNPs very valuable for pharmaceutical, cosmetic, food industry applications, and antimicrobial treatments [22,23,24].

The potential of AgNPs to address ecological challenges is currently being studied, especially for inhibiting cyanobacterial overgrowth in water bodies like Yahuarcocha Lagoon (Ecuador) [25,26]. Cyanobacteria, commonly known as blue-green algae, are ubiquitous in freshwater environments such as lakes and lagoons, often thriving in proximity to human populations [27,28]. Several water bodies worldwide have reported cyanobacterial blooms, including Winnipeg Lake (Canada) [29] and the Indian River Lagoon (USA) [30]. The Yahuarcocha Lagoon exhibits alarmingly high cyanobacterial concentrations, reaching 414 ppb [31].

Cyanobacteria can produce an array of cytotoxins, including microcystins, anatoxins, saxitoxins, cylindrospermopsins, hepatotoxins, and neurotoxins, which pose severe health risks to both humans and animals [32]. These microorganisms can be cultivated under controlled laboratory conditions using nutrient-rich media such as Z8 medium [33,34], which consists of NaNO_3_, Ca(NO_3_)_2_, EDTA, and trace elements. However, cyanobacterial growth is highly sensitive to environmental factors, including salinity, pH, light intensity, and temperature [35].

Silver nanoparticles are known to exert an oligodynamic effect, a biocidal mechanism against bacteria. Several factors, such as nanoparticle size, surface charge, shape, and surface coating, significantly influence their antibacterial efficacy [36]. Although the precise mechanism of AgNP-induced antibacterial activity remains under investigation, existing evidence suggests that AgNPs directly interact with bacterial cell membranes, inducing oxidative stress via active oxygen species, disrupting ATP synthesis, and ultimately inhibiting DNA replication [37]. Other studies propose that AgNPs generate reactive oxygen species (ROS), leading to biomolecular damage and bacterial cell death [38,39].

The green synthesis of AgNPs using plant extracts has garnered increasing interest due to its eco-friendly, cost-effective, and scalable nature. Several plant species have been utilized as reducing agents in AgNP synthesis, including *Camellia sinensis* (green tea) [40], *Datura metel* [41], *Diospyros kaki* [42], *Nelumbo nucifera* [43], *Capsicum annuum* [44], and *Aloe vera* [34]. The reduction of silver ions Ag^+^ into AgNPs is typically facilitated by polyphenols and flavonoids present in these plant extracts [45].

*Aloe vera*, a widely known medicinal plant, consists of three distinct layers: an inner gel-like layer composed mainly of water along with amino acids, lipids, sterols, glucomannans, and vitamins; a middle layer containing bitter yellow sap rich in glycosides and anthraquinones; and an outer protective layer [46,47]. *Aloe vera* contains lignin, hemicellulose, and aloin molecules, which can effectively reduce silver ions to form AgNPs [34].

Recent studies (2023–2025) have emphasized the potential of plant-mediated silver nanoparticles for controlling microbial systems, including cyanobacteria and microalgae in aquatic environments. Green-synthesized AgNPs derived from plant extracts such as Aloe vera, Camellia sinensis, and other phytochemical-rich sources have demonstrated enhanced biocompatibility, reduced toxicity, and tunable antibacterial properties. However, most studies have focused on common bacterial strains, with limited attention to freshwater cyanobacterial blooms, particularly in high-altitude ecosystems such as Andean lagoons. Therefore, this work addresses a critical gap by evaluating the antibacterial performance of Aloe vera-mediated AgNPs against cyanobacteria isolated from the Yahuarcocha Lagoon, providing both local environmental relevance and a scalable green nanotechnology approach [48,49,50].

This study targets the synthesis of AgNPs using *Aloe vera* extract, silver nitrate solution, and polyvinylpyrrolidone as a stabilizing agent. Furthermore, the minimum inhibitory concentration of the AgNP solution was determined in samples containing Z8 medium and cyanobacteria. The results of this study show the potential of synthesizing environmentally sustainable nanomaterials for implementation in future water treatment and biotechnological applications.

The remainder of the paper is organized as follows: Section 2 describes the materials and methods used in the study, including the green synthesis of AgNPs using *Aloe vera* extract, the optimization of pH and light conditions, and the characterization techniques such as UV–Vis spectroscopy and SEM.

## 2. Materials and Methods

### 2.1. Aloe vera Extract Preparation

The *Aloe vera* extract was prepared using fresh leaves thoroughly washed with distilled water to remove any surface contaminants. The leaves were cut into small pieces to facilitate pulp extraction, and 20 g of pulp was obtained by manually crushing the pieces using a mortar and pestle.

The extraction process involved heating 20 g of *Aloe vera* pulp with 200 mL of H_2_O in a 250 mL beaker at 25 °C for 35 min, followed by a resting period of 15 min to allow for complete extraction. Finally, the solution was filtered using filter paper to remove any remaining solid particles. The extract served as the reducing agent in the green synthesis of silver nanoparticles (AgNPs).

### 2.2. Silver Nanoparticle Solution Preparation

To synthesize the silver nanoparticle solution, 0.092 g of silver nitrate (AgNO_3_) and 0.09 g of polyvinylpyrrolidone (PVP, 10 kDa molecular weight) were separately placed in a beaker. Subsequently, 90 mL of distilled water was added, and the mixture was stirred until complete dissolution of the granules was achieved.

**Figure 1:**
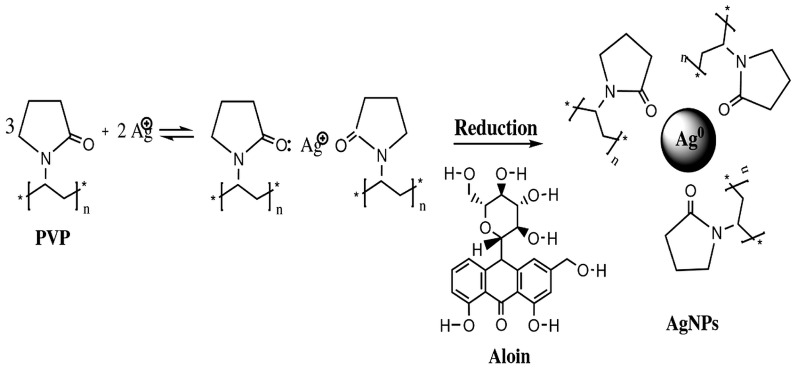
Schematic representation of the interaction between polyvinylpyrrolidone (PVP) and aloin with silver ions (Ag^+^), leading to the formation of silver nanoparticles (AgNPs). Asterisks indicate repeating or continuation points in the PVP polymer chain.

A pH-adjusted aqueous solution was produced dropwise using a 1 mM sodium hydroxide (NaOH) solution until reaching the desired values of 7, 8, and 8.45. The pH of the solution was measured using a calibrated bench pH meter (HI2020-01, Hanna Instruments, Woonsocket, RI, USA), following standard calibration procedures. Silver nanoparticles were then synthesized by introducing 10 mL of *Aloe vera* extract into the final solution. The reaction mixture was maintained under a 2800 lux LED lamp for 20 min to facilitate nanoparticle formation. The chemical reaction occurring during this process is illustrated in Figure 1.

The silver concentration was estimated based on the initial amount of AgNO_3_ used in the synthesis. Considering the molar mass ratio between Ag and AgNO_3_, approximately 63.5% of the precursor mass corresponds to elemental silver. This estimation assumes complete conversion and no material losses, and therefore represents a nominal upper-bound concentration. The actual distribution between nanoparticle-bound silver and dissolved Ag^+^ was not determined.

### 2.3. Ultraviolet–Visible (UV–Vis) Spectroscopy

The synthesized AgNPs using *Aloe vera* extract, polyvinylpyrrolidone (PVP, 10 kDa molecular weight), and AgNO_3_ were characterized using UV-Vis spectroscopy (SPECORD S 600, Analytik Jena GmbH+Co. KG, Jena, Germany). Wavelengths in the range of 300–800 nm were recorded from the absorption spectra to confirm the presence and stability of AgNPs.

### 2.4. Scanning Electron Microscopy (SEM)

Scanning electron microscopy (SEM) was utilized (Inspect F50, FEI Company, Hillsboro, OR, USA) to identify the morphological characteristics and size distribution of AgNPs. Sample preparation involved placing a drop of the solution onto a piece of carbon tape, which was then allowed to air-dry for 48 h before being placed into the microscope chamber. Observations were conducted using an accelerating voltage ranging from 5 kV to 15 kV, with magnification adjustments applied to enhance resolution for optimal nanoparticle visualization.

### 2.5. Silver Nanoparticle Antibacterial Tests

The experimental campaign consists of 18 samples to assess the antibacterial efficacy of AgNPs against cyanobacteria. Each sample contained 6 mL of Z8 liquid medium and 0.5 mL of cyanobacteria culture. The AgNP solution was added incrementally, starting with 10 μL, increasing by 10 μL increments up to 100 μL, and subsequently increasing by 100 μL increments up to a maximum volume of 900 μL. The inhibitory effect of AgNPs on cyanobacterial growth was then evaluated.

### 2.6. Fluorescence Microscopy Setup

A Leica DM4000 B LED fluorescence microscope (Leica Microsystems, Wetzlar, Germany) was employed to examine the morphological characteristics of cyanobacteria. Three different fluorescence filters (Y5, N21, and I3) were utilized, with their respective excitation (EX) and emission (EM) wavelengths detailed in Table 1.

### 2.7. Total Dissolved Solids (TDS) Measurement

The TDS content was measured two months after introducing the algae culture using a Hanna HI98192 meter (Hanna Instruments, Woonsocket, RI, USA) to evaluate the effect of AgNPs on water quality.

### 2.8. Data Analysis

The experimental design aimed to analyze the relationship between AgNP concentration, algae concentration (absorbance at 630 nm), and their interaction. Turbidity was set as the response variable for linear regression analysis and ANOVA.

To improve model fitting, the AgNP concentration ([AgNPs]) data were transformed using the reciprocal function (1/x) before applying the regression model, as described in Equation (1).

(1) $$\log\left(\mathrm{Abs}\right)=a+\frac{b}{x}$$

where Abs denotes absorbance at 630 nm, $x=[\begin{array}{c} \mathrm{AgNPs} \end{array}]$ is the AgNP concentration, and *a* and *b* are fitted coefficients.

Model validation was assessed based on residual plots, effect size, and *p*-values, with a significance threshold of α=0.05. All statistical analyses were conducted using RStudio (version 2023.09.1+494; Posit Software, PBC, Boston, MA, USA).

## 3. Results and Discussion

### 3.1. Ultraviolet–Visible Spectrophotometry

The UV–Vis spectra of silver nanoparticles showed distinctive characteristics directly related to their size, shape, and surface chemistry. The observed surface plasmon resonance (SPR) peaks arise due to the collective oscillation of free electrons in response to incident light, a key feature in the optical properties of AgNPs. In this study, AgNPs were synthesized using silver nitrate (AgNO_3_) as the precursor, *Aloe vera* extract as the reducing agent, and polyvinylpyrrolidone (PVP, 10 kDa MW) as the stabilizer. Mardkour [51] and Zein [52] described the role of PVP in the synthesis process, stating that PVP adheres to the nanoparticle surface while its polymer chains enclose and stabilize the particles.

Several experimental parameters were tested to optimize AgNP formation, including: AgNO_3_ concentration, PVP concentration, the percentage of *Aloe vera* extract, LED exposure time, and synthesis conditions under different temperatures. Based on the results, the optimal synthesis conditions are 6 mM AgNO_3_, 0.1% (*w*/*v*) PVP (10 kDa MW), 10% *Aloe vera* extract, and 20 min of exposure to a 2800 lux LED lamp.

**Figure 2:**
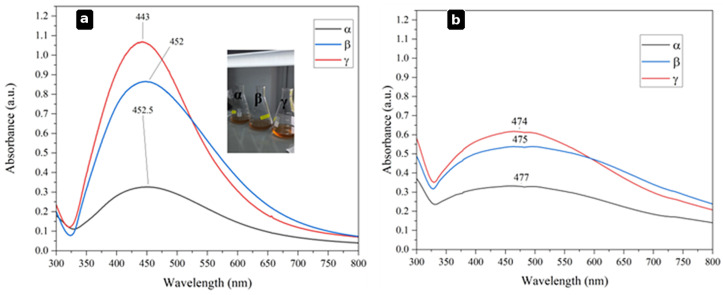
UV–Vis spectra of AgNPs synthesized with *Aloe vera* extract and PVP: (**a**) freshly synthesized samples; (**b**) samples after 60 days of storage at 4 °C. Samples: (α) pH 7.0, (β) pH 8.0, and (γ) pH 8.45.

Results showed that SPR peaks appeared between 425 and 460 nm, with slight variations depending on the pH of the synthesis solution (Figure 2a). AgNPs synthesized at pH 8 showed a maximum SPR peak at 452 nm, whereas the same solution with pH 8.45 revealed a minimal blue shift to 443 nm. Similarly, AgNP solutions prepared at neutral pH showed peak values at 452.5 nm; still, the intensity of this peak is lower compared to those prepared at higher pH.

The experimental data agree with the previous findings of Kaur et al. (2021), where SPR readings follow a similar response with changes in pH, clearly highlighting that synthesis conditions significantly influence the plasmonic properties of AgNPs [53,54,55,56].

An SPR shift similar to that observed in this research was previously reported by several authors, including Bindhu and Umadevi [57], who synthesized AgNPs using *Ananas comosus* extract. Authors like Yallappa [58] and Vélez [59] pointed out the critical role of the SPR peak position with the concentration of plant extract. In this study, the influence of the reducing agent concentration on the optical properties of AgNPs was confirmed by SPR peak measurements, which ranged between 400 and 500 nm. Additionally, findings also indicate that alkaline conditions (pH>7) were favorable for the formation and stability of AgNPs, a phenomenon also reported in previous studies by Nadzir [60] and Rodríguez-León [61]. Among the synthesized samples (α, β, and γ), the γ sample exhibited the highest absorbance, suggesting a higher concentration of AgNPs under these conditions.

Further spectral analysis after 60 d of storage at 4 °C revealed significant red shifts in SPR peaks, moving toward longer wavelengths (470–480 nm) (Figure 2b). This shift suggests alterations in AgNP properties over time, most likely due to particle aggregation, shape modifications, or oxidation effects.

- Sample α (initial peak at 452.5 nm) shifted to 477 nm.
- Sample β (initial peak at 452 nm) shifted to 475 nm.
- Sample γ (initial peak at 443 nm) shifted to 474 nm.

As shown in the results, the spreading of absorbance peaks suggests that numerous variables contribute to AgNP instability over extended storage. Previous studies reported similar observations, indicating that changes in particle size, shape, and potential agglomeration contribute to the broadening and shifting of SPR peaks [62,63]. Additionally, researchers have emphasized that AgNPs are highly susceptible to oxidation, impacting their stability and hydrolysis processes, which can degrade PVP, ultimately influencing the structural integrity and optical properties of AgNPs [64,65].

The observed shift in the SPR band with increasing pH can be attributed to changes in nanoparticle size, surface charge, and local dielectric environment. At higher pH values, deprotonation of functional groups present in the Aloe vera extract enhances their interaction with silver ions and nanoparticle surfaces, influencing nucleation and growth processes. This may lead to variations in particle size distribution and aggregation state, which directly affect the position and intensity of the SPR band.

Additionally, changes in surface charge at alkaline pH can improve electrostatic stabilization, modifying interparticle interactions and optical response. The surrounding dielectric environment, determined by adsorbed phytochemicals and stabilizing agents, also plays a key role in defining the plasmonic behavior of the system.

Our results strongly suggest that changes in nanoparticle size, shape, and spatial distribution, most likely triggered by oxidation of silver surfaces and gradual aggregation of particles, are the primary contributors to the broadening and red-shifting of the surface plasmon resonance (SPR) peaks observed after extended storage at 4 °C. Specifically, the SPR peaks of samples α, β, and γ initially located at 452.5 nm, 452 nm, and 443 nm shifted to 477 nm, 475 nm, and 474 nm, respectively, after 60 d, indicating a consistent 20–30 nm red shift. This behavior is consistent with previous reports describing how nanoparticle growth or agglomeration leads to a decrease in electron density and a consequent shift toward longer wavelengths [62].

The broadening of the absorption bands further supports the hypothesis that a wider size distribution was present after storage, likely caused by partial fusion of nanoparticles or destabilization of the PVP coating, as reported by Amiri [65]. Notably, the γ solution (pH 8.45) not only exhibited the most intense SPR peak among the three samples, but also retained a relatively narrow spectral profile compared to α and β, suggesting a higher colloidal stability and a greater concentration of well-dispersed AgNPs under alkaline conditions. For these reasons, the γ solution was selected for subsequent antibacterial assays, ensuring that the experimental tests were performed with the most concentrated and stable nanoparticle system, thus providing reliable and reproducible inhibitory activity data.

It is worth mentioning that although plant extracts are widely recognized for their ability to act as reducing, capping, and stabilizing agents in green nanoparticle synthesis, the incorporation of PVP in this study was intended as a supplementary stabilizing agent. The complex and variable composition of Aloe vera extract can lead to differences in nanoparticle nucleation, growth, and aggregation behavior. Therefore, PVP was introduced to enhance colloidal stability, reduce aggregation, and improve reproducibility under the specific synthesis conditions employed.

To further establish the relationship between synthesis conditions and the resulting nanoparticle properties, a correlation summary is presented in Table 2.

This correlation highlights how key synthesis parameters influence nanoparticle formation and stability, supporting the observed experimental trends. Notably, alkaline pH and the presence of PVP appear to play an important role in controlling nanoparticle stability and dispersion.

### 3.2. Scanning Electron Microscopy (SEM) and Energy-Dispersive X-Ray Spectroscopy (EDX)

**Figure 3:**
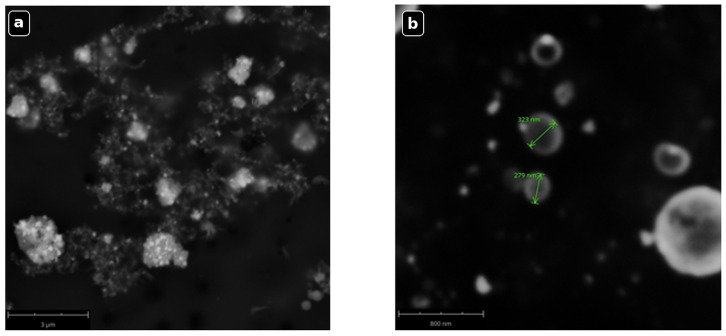
SEM images of AgNPs synthesized using the green synthesis method: (**a**) image captured at 22,000× magnification and (**b**) image captured at 87,000× magnification.

SEM was used to study the morphology and size distribution of the synthesized silver nanoparticles. In Figure 3a,b, the AgNPs show a spherical structure with particle sizes between 300 and 500 nm. Uniform distribution of nanoparticles suggests effective dispersion and stabilization at synthesis. Additionally, the SEM images exposed key characteristics of AgNPs’ surface texture, such as porous, bright, and rough characteristics (Figure 3b). These results confirm the successful synthesis of AgNPs with the desired morphology, consistent with previous studies [66,67].

AgNO_3_ concentration, pH, temperature, and the presence of biomolecules such as polysaccharides, phenolic compounds, and aloin groups from the *Aloe vera* extract could influence the particle size of AgNPs, which is fundamental in the reduction and stabilization of nanoparticles [68,69,70].

The SEM analysis provided insights into the morphology and size distribution of the synthesized AgNPs, revealing predominantly spherical particles with diameters ranging from 300 to 500 nm and a relatively uniform spatial distribution. The porous and rough surface texture observed under high magnification suggests the presence of surface-bound phytochemicals from the *Aloe vera* extract, which may act as capping agents and contribute to nanoparticle stabilization.

Interestingly, the particle sizes reported here are considerably larger than those in several previous green-synthesis studies, where average AgNP diameters ranged from approximately 2.49 to 200 nm [59,71,72,73,74]. This size discrepancy could be attributed to several synthesis parameters, including the relatively high pH (8.45), the specific molecular weight of PVP (10 kDa), and the concentration of *Aloe vera* extract, all of which are known to influence nucleation and growth kinetics during AgNP formation. Larger particle sizes may also result from slower nucleation rates or partial aggregation during the synthesis or drying process. Additionally, partial aggregation during drying for SEM analysis may contribute to the apparent increase in particle size.

**Figure 4:**
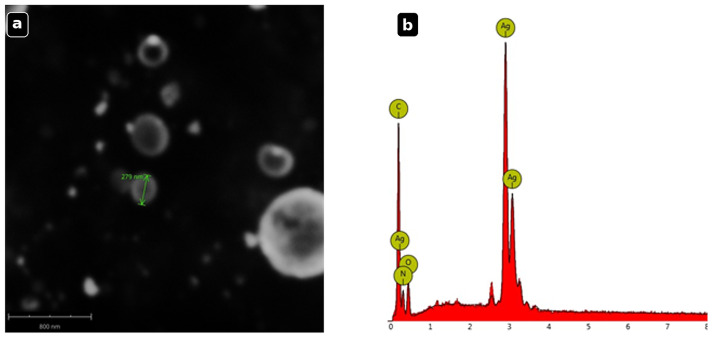
EDX spectrum indicating the elemental composition of AgNPs: (**a**) SEM image at 87,000× magnification and (**b**) EDX spectrum showing the elemental composition of AgNPs. The complementary EDX analysis confirmed the chemical identity of the nanoparticles, showing a dominant silver signal (59.96 wt%), along with minor contributions from carbon, oxygen, and nitrogen. The detection of C, O, and N is consistent with the presence of plant-derived organic moieties and residual PVP molecules adsorbed on the nanoparticle surface, which is desirable as these covering layers enhance colloidal stability.

To further confirm the chemical composition of the synthesized AgNPs, Energy-Dispersive X-ray Spectroscopy (EDX) analysis was conducted. As shown in Figure 4, the EDX spectrum exhibited a strong signal corresponding to silver (Ag), confirming its presence as the primary component. Additionally, peaks corresponding to carbon (C), oxygen (O), and nitrogen (N) were detected, which can be attributed to the following:

- Carbon (C): This likely originates from the carbon tape used for sample mounting.
- Oxygen (O) and nitrogen (N): Possibly due to surface contaminants or residual biomolecules from the *Aloe vera* extract.

Together, the SEM and EDX results not only validate the successful green synthesis of AgNPs but also provide evidence of a bio-organic surface coating that could be important in their antibacterial performance, making these nanoparticles suitable for eco-friendly antimicrobial and water treatment technologies.

### 3.3. Fluorescence Microscopy Observations

After a two-month incubation period, the control samples were analyzed using a physiological saline solution to improve clarity in observing the morphological characteristics of cyanobacteria under a fluorescence microscope. The fluorescence analysis provided insights into the pigment composition and structural integrity of cyanobacteria grown in Z8 medium.

**Figure 5:**
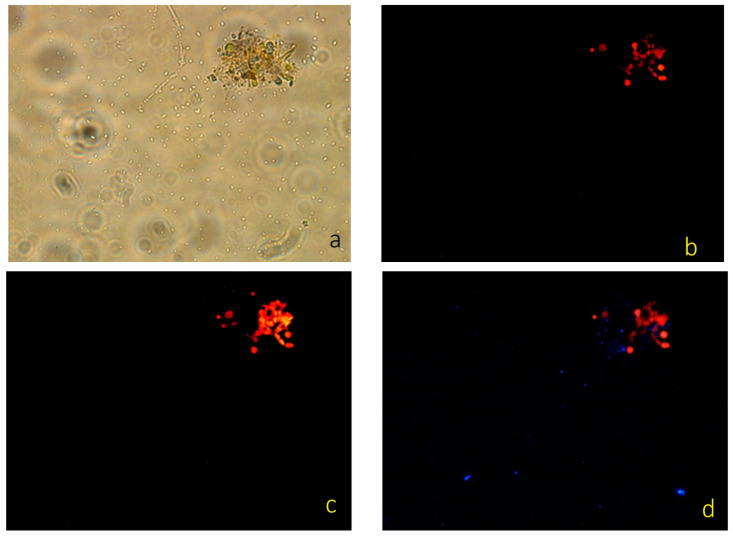
Fluorescence microscopy images of cyanobacteria cultured in Z8 medium: (**a**) control sample viewed at 40× magnification, contrast enhancement (40%); (**b**) control sample viewed at 40× magnification using the Y5 filter (EX: 590–650 nm, EM: 662–738 nm); (**c**) control sample viewed at 40× magnification using the N21 filter (EX: 515–561 nm, EM: 590 nm); (**d**) control sample viewed at 40× magnification using the I3 filter (EX: 450–490 nm, EM: 515 nm).

Figure 5a shows a cluster of blue-green algae, with cyanobacterial colonies exhibiting a rounded morphology. The characteristic blue-green hue is attributed to the presence of chlorophyll *a*, which plays a crucial role in photosynthetic activity. Figure 5b demonstrates the fluorescence of chlorophyll *a*, observed within the excitation (EX) range of 590–650 nm and emission (EM) range of 662–738 nm. The relatively low fluorescence intensity suggests a moderate concentration of pigment–protein complexes within the cyanobacterial cells.

In Figure 5c, fluorescence excitation at 515–561 nm with an emission peak at 590 nm indicates the predominant excitation of fluorophores in this spectral range. This is consistent with previously reported fluorescence emission profiles of chlorophyll *a* in cyanobacteria, where specific excitation wavelengths trigger distinct fluorescence responses. Figure 5d, which employs the I3 filter, reveals the presence of faint red fluorescence associated with chlorophyll *a*, alongside a subtle blue fluorescence signal (EX: 450–490 nm, EM: 515 nm). The appearance of blue fluorescence is likely due to trace elements present in the Z8 medium, which may have interacted with cyanobacterial pigment–protein complexes.

These observations are consistent with Sarcina and Mullineaux [75], who demonstrated that chlorophyll *a* in cyanobacteria exhibits fluorescence across multiple excitation wavelengths (405, 476, 488 and 496 nm). Their findings suggest that pigment–protein complexes absorb light within this range, leading to fluorescence emission at various stages of the energy transfer chain. The results in Figure 5d closely align with these findings, reinforcing that chlorophyll *a* fluorescence is primarily localized within the thylakoid membranes of cyanobacteria.

Overall, these fluorescence microscopy observations not only confirmed the structural integrity and spatial distribution of pigments within cyanobacterial cells grown in Z8 medium but also provided evidence of active photosynthetic machinery. The presence of intense chlorophyll *a* fluorescence across multiple excitation/emission filter sets suggests that thylakoid membranes remain intact and functional, which is essential for light harvesting and energy conversion. This finding is consistent with previous studies reporting that cyanobacteria cultured in nutrient-rich media exhibited robust pigment–protein complex organization and maintained high photosynthetic efficiency [75].

The detection of additional blue and red fluorescence signals may also indicate the presence of accessory pigments, such as phycobiliproteins, which contribute to light absorption in different spectral regions. Importantly, confirming the physiological health of the cyanobacterial culture establishes a reliable baseline for subsequent antibacterial assays with AgNPs, ensuring that any observed growth inhibition can be attributed to nanoparticle treatment rather than pre-existing cellular stress or pigment degradation.

Fluorescence microscopy analysis was performed to qualitatively assess the presence and integrity of photosynthetic pigments in cyanobacterial cultures. The observed fluorescence signal in control samples is consistent with chlorophyll-associated emission, confirming the viability of untreated cultures. However, no quantitative fluorescence analysis or viability-specific staining (e.g., Live/Dead assays) was conducted. Additionally, fluorescence data from treated samples were not systematically analyzed for comparison. Therefore, the fluorescence results should be interpreted as preliminary and qualitative observations, providing supportive but not conclusive evidence regarding the biological state of the system. Further studies incorporating quantitative fluorescence techniques and viability assays would be required to elucidate the mechanisms of inhibition in greater detail.

### 3.4. Antibacterial Effect of Silver Nanoparticles

**Figure 6:**
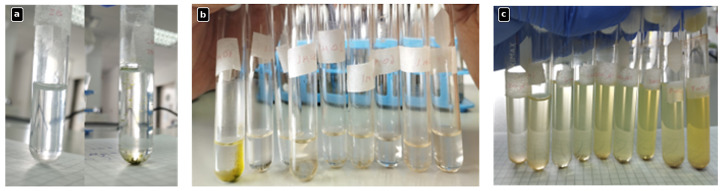
Antibacterial effect of AgNPs synthesized using green synthesis: (**a**) control sample (untreated cyanobacteria); (**b**) samples treated with AgNPs ranging from 10 to 90 μL; (**c**) samples treated with AgNPs ranging from 100 to 900 μL.

The antibacterial efficacy of the silver nanoparticles was assessed in Z8 medium containing cyanobacteria. As shown in Figure 6, the antibacterial impact was evaluated over a two-month period following AgNP treatment, using incremental volumes of 10 μL (Figure 6b) and 100 μL (Figure 6c).

The results demonstrate a concentration-dependent antibacterial effect of AgNPs on cyanobacteria growth. At lower AgNP concentrations (Figure 6b), partial inhibition of bacterial growth was observed. However, at higher AgNP concentrations (Figure 6c), a significant reduction in bacterial density was evident, indicating a concentration–response relationship.

During the experimental period, bacterial growth was detected only in the test tube containing 10 μL of AgNPs, while all other samples exhibited complete bacterial inhibition. To determine the Minimum Inhibitory Concentration (MIC) necessary to prevent bacterial growth, the γ solution (578 mg/mL AgNPs) was used as a reference. The lowest concentration at which no bacterial growth was observed was 20 μL of AgNPs, corresponding to an MIC value of 1.77 mg/mL.

Determining MIC values is crucial, as it provides information on the antibacterial potential of AgNPs and their prospective applications in biomedical and environmental settings. The MIC observed in this study was compared with previous antibacterial studies utilizing AgNP treatments. For instance, Logaranjan [67] tested AgNPs against *Bacillus cereus*, *Escherichia coli*, and *Staphylococcus aureus*. Burange [73] examined their effects on *Salmonella typhi* and *Bacillus subtilis*. Vélez [59] and Oukarroum [76] investigated the antibacterial effects of AgNPs on *Kocuria* variants and *Dunaliella tertiolecta*, respectively.

When comparing our MIC value (1.77 mg/mL) with those reported in prior studies by Dong [77] and Oukarroum [76] (1 mg/mL and 0.01 mg/mL, respectively), it is evident that our calculated MIC is higher. This difference may be attributed to variations in bacterial strains, AgNP synthesis conditions, and experimental setups. Factors such as AgNP size, shape, surface charge, and environmental parameters (pH, ionic strength, and temperature) can significantly influence MIC values.

Although reactive oxygen species (ROS) were not directly quantified in this study, the observed concentration-dependent inhibition and exponential decay behavior strongly support an oligodynamic effect mechanism. It is well established that AgNPs can induce oxidative stress, disrupt membrane integrity, and interfere with cellular respiration and DNA replication. The consistent reduction in absorbance and complete inhibition at relatively low volumes suggest that nanoparticle–cell interactions play a dominant role, likely mediated by both surface-bound silver and released Ag^+^ ions. Future studies should incorporate ROS quantification and viability staining to further validate these mechanisms.

The antibacterial assessment performed in this study was designed as an exploratory evaluation of growth inhibition under prolonged exposure conditions rather than a standardized microbiological MIC assay. The extended incubation period (up to two months) was intentionally selected to assess long-term inhibitory behavior and system stability under conditions that more closely resemble environmental exposure scenarios in aquatic systems. While conventional MIC assays are typically conducted over short incubation periods (24–72 h), the longer duration employed here enables the evaluation of nanoparticle stability, aggregation behavior, and sustained biological interaction over time. Despite the absence of control experiments using silver ions, stabilizing agents, and Aloe vera extract alone—which limits the ability to fully differentiate between nanoparticle-specific effects and other contributing factors such as ion release or extract-derived activity—the results show clear concentration-dependent inhibition trends. All experiments were conducted under consistent laboratory conditions to minimize variability; however, further studies incorporating biological replicates and standardized microbiological protocols are required to improve statistical robustness. Overall, the findings provide insight into the interaction between AgNP-based systems and cyanobacterial cultures under sustained exposure conditions, although they should be interpreted within the exploratory scope of the present study.

### 3.5. Total Dissolved Solids (TDS) Analysis

TDS is a key metric quantifying the total organic and inorganic content dissolved in a liquid. After a two-month period following AgNP addition, the absorbance at 630 nm and TDS values (mg/L) were measured to assess potential changes in the chemical composition of the medium. These measurements provide further insights into the impact of AgNPs on cyanobacteria and water quality parameters.

**Figure 7:**
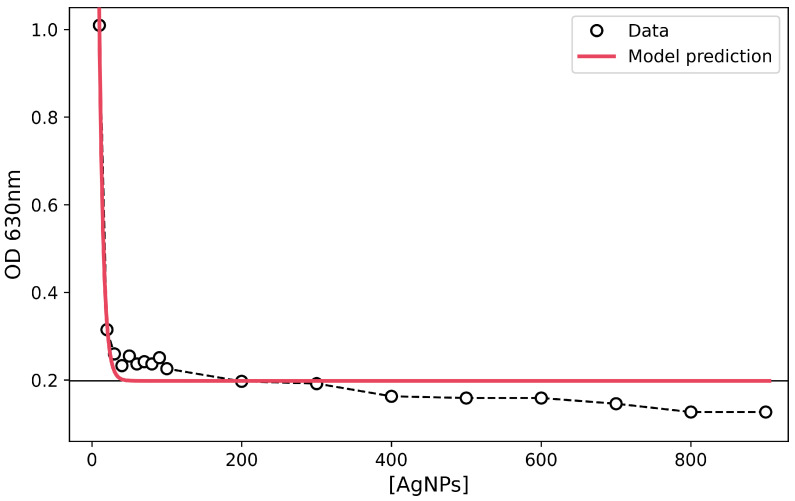
Effect of AgNP treatment (10–90 μL) on absorbance at 630 nm, representing bacterial growth inhibition.

The effect of AgNP treatment (ranging from 10 to 900 μL) on bacterial inhibition was analyzed using absorbance measurements and a concentration–response model (Figure 7). The results demonstrate a correlation between AgNP concentration and bacterial growth, with the highest peak observed at 10 μL of AgNPs. As the AgNP concentration increased, bacterial growth significantly decreased, indicating a strong inhibitory effect on algae proliferation.

The observed trend suggests a concentration-dependent inhibition, where higher AgNP concentrations effectively suppress cyanobacterial growth. This trend aligns with previous studies demonstrating the antimicrobial properties of AgNPs against various bacterial species.

Furthermore, Total Dissolved Solids (TDS) data were used to develop a concentration–response model in RStudio, where the variables included: algae concentration, AgNP concentration, and TDS levels (mg/L). Using the data from Table 3, an exponential function was obtained to describe the relationship between AgNP concentration and algae removal efficiency.

The statistical model represents the effectiveness of AgNPs in algae removal, with the coefficient in Equation (1) indicating that at approximately 1000 μL, the optical density (OD) is reduced to 0.16, suggesting complete inhibition of algae growth.

The concentration-dependent inhibition pattern is visually represented in Figure 7 and further validated by the statistical results presented in Table 3. The proposed method appears to effectively reduce algae concentration within a short time frame, following an exponential decay model, as highlighted in the model fitting summary. Data supported first-order kinetics behavior for the TDS reduction; this reduction kinetic points out a great potential for these nanoparticles to be tested at industrial scales. However, the scalability of the technology must be tested in future research.

**Figure 8:**
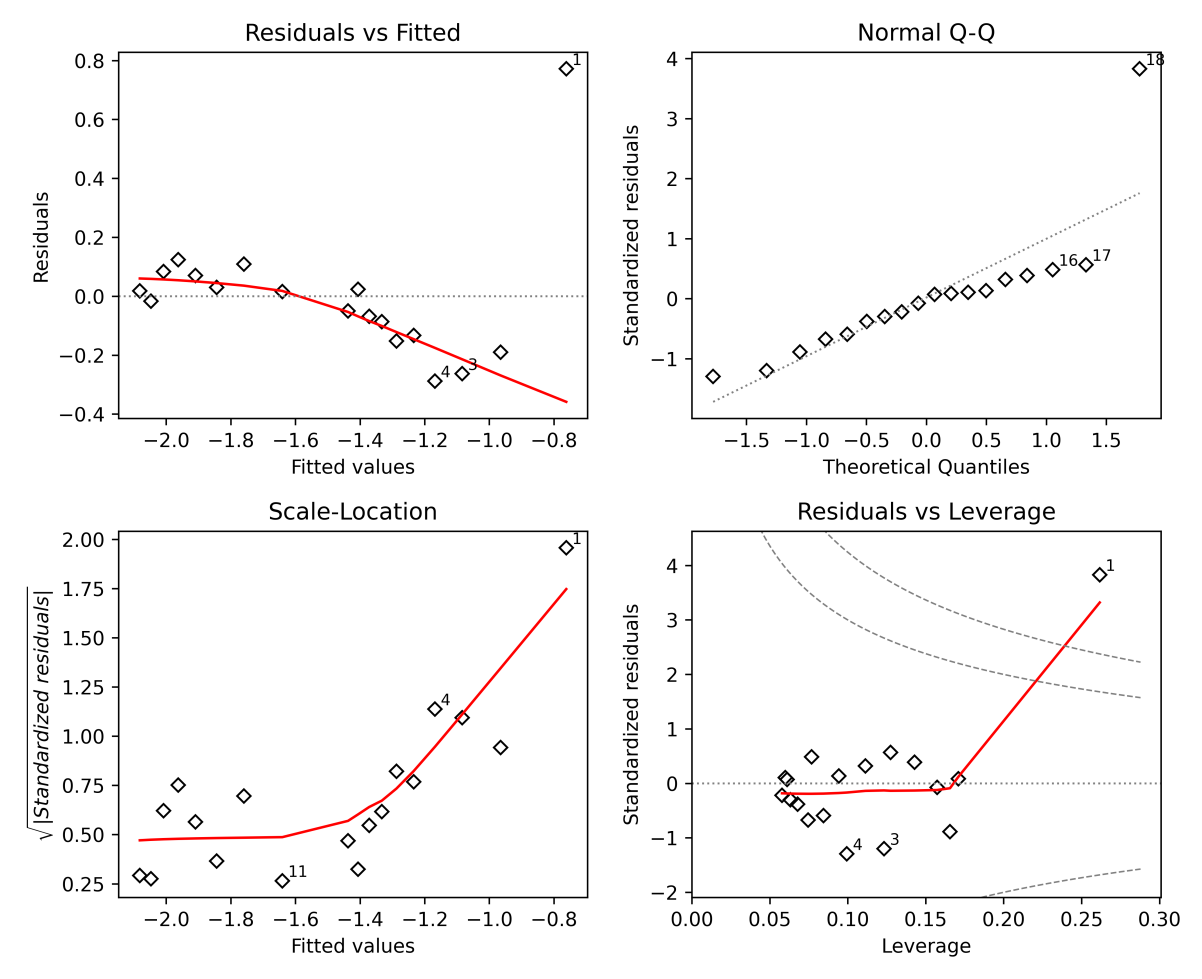
Validation plot and residual analysis of the concentration–response model using RStudio. Black diamonds represent observed residuals, red curves indicate the smoothed trend, and gray dashed lines indicate reference lines, including the zero-residual line, the normal Q–Q reference line, and Cook’s-distance contours where applicable.

Figure 8 presents the validation of the concentration–response model in RStudio, confirming that the data fit an exponential decay pattern. This model effectively demonstrates the relationship between AgNP concentration and bacterial inhibition, further supporting the practical application of AgNPs for microbial control in water treatment and biomedical applications, and provides critical information for scalability studies under standard conditions.

To provide a concise overview of the characterization results obtained through different analytical techniques, a summary is presented in Table 4.

This integrated view highlights the consistency between structural, optical, and antibacterial properties of the synthesized nanoparticles. Additional supporting data are provided in the Supplementary Materials.

### 3.6. Limitations

This study has several limitations that should be considered when interpreting the results. Particle size analysis was performed using SEM, which may not distinguish between primary nanoparticles and aggregates formed during drying. In addition, the silver concentration was estimated from precursor quantities and was not independently quantified. The antibacterial assays were conducted without individual component controls (AgNO_3_, PVP, and *Aloe vera* extract), which limits the ability to attribute the observed effects to a specific component. These limitations define the scope of the present work as exploratory.

## 4. Sustainability and Scalability

From a sustainability perspective, the proposed green synthesis route offers significant advantages over conventional chemical reduction methods, including reduced use of toxic reagents and lower environmental impact. However, the potential release of silver ions into aquatic systems remains a concern and should be carefully evaluated in future studies. Life-cycle assessment approaches will be essential to fully understand the environmental footprint and long-term implications of applying AgNPs in freshwater ecosystems.

Although large-scale production was not explored in this study, the simplicity of the synthesis process, low-cost raw materials, and mild reaction conditions suggest strong potential for scalability. Future work should focus on quantifying production yield, cost per unit mass, and process optimization under industrial conditions.

## 5. Conclusions

This study presents an exploratory, application-oriented evaluation of green-synthesized silver-based nanostructures in an environmentally relevant context.

Silver-based nanostructures were synthesized using a green approach involving *Aloe vera* extract, silver nitrate, and polyvinylpyrrolidone (PVP) as the reducing, precursor, and stabilizing agents, respectively. The synthesis was conducted at pH 8.45, adjusted with a 1 mM NaOH solution, and carried out under LED illumination (2800 lux for 20 min), which promoted the formation of silver-containing structures, as indicated by observable color changes.

The formation of these structures was supported by UV–Vis spectroscopic analysis, where a surface plasmon resonance (SPR) band in the range of 425–460 nm was observed. SEM images revealed predominantly spherical features in the 300–500 nm range; however, it was not possible to conclusively distinguish primary nanoparticles from aggregates using this technique alone. Higher absorbance at pH 8.45 suggests that pH plays an important role in the synthesis process. Additionally, the presence of phenolic compounds, polysaccharides, and aloin in the *Aloe vera* extract is likely to contribute to the reduction and stabilization processes.

The antibacterial response of the system was evaluated in Z8 medium containing cyanobacteria, showing a reduction in growth with increasing dosage. A nominal minimum inhibitory concentration (MIC) of 1.77 mg/mL was estimated based on precursor input.

Overall, the results indicate potential antibacterial behavior of the system under the tested conditions. Nevertheless, further studies are required to confirm particle characteristics, quantify silver content, include more test controls, and elucidate the mechanisms governing the observed effects. These aspects will be addressed in future work to validate the applicability of this approach in water treatment systems.

## Figures and Tables

**Table 1:** Fluorescence filters and their corresponding wavelengths.

Filters	Excitation (EX) [nm]	Emission (EM) [nm]
Y5	590–650	662–738
N21	515–561	590
I3	450–490	515

**Table 2:** Correlation between synthesis parameters and AgNP properties.

Parameter	Condition Tested	Observed Effect
pH	7–8.45	Higher pH improved SPR intensity and stability
Aloe vera extract	10% (*v*/*v*)	Effective reduction and capping
PVP (10 kDa)	0.1% (*w*/*v*)	Improved dispersion and stability
LED exposure	2800 lux, 20 min	Enabled nanoparticle formation
Temperature	Heating step	Enhanced extraction of reducing agents
Outcome	—	Stable AgNPs with antibacterial activity

**Table 3:** Linear Regression Results for Two-Way ANOVA on Bacterial Growth Inhibition.

Variables	Mean	SEM	*t*-Value	*p*-Value
Intercept (*a*)	1.81206	6.01	29.419	<0.001
$1/[\begin{array}{c} \mathrm{AgNPs} \end{array}]$ (*b*)	−17.15956	0.0093	−10.509	<0.001
Effect size	0.87 (*p*-value = $1.923\times 10^{-8}$)			
F-ratio (DF)	105.7 on 1 and 16 DF			

**Table 4:** Summary of characterization techniques and key findings.

Technique	Parameter Evaluated	Key Findings
UV–Vis	SPR peak position	425–460 nm (fresh), 470–480 nm (60 days)
SEM	Morphology and size	Spherical particles, 300–500 nm
EDX	Elemental composition	Ag: 59.96 wt%, C, O, N traces
Fluorescence	Pigment integrity	Chlorophyll fluorescence preserved in control samples
Antibacterial	MIC and inhibition	MIC = 1.77 mg/mL, complete inhibition ≥ 20 μL

## Data Availability

The data presented in this study is available in Zenodo at https://doi.org/10.5281/zenodo.18687000. The repository includes the UV–Vis absorption spectra, antibacterial concentration–response data, exponential decay model outputs, and regression diagnostic datasets supporting the statistical validation of the model.

## Supplementary Materials

The following supporting information can be downloaded at: https://www.mdpi.com/article/10.3390/life16071132/s1, S1: Purpose and Scope of This Supplementary Document; S2: Functional Roles of Each Component in the Synthesis System; S3: Why *Aloe vera* Extract Is Not the Antibacterial Agent; S4: Why Polyvinylpyrrolidone (PVP) Is Not the Antibacterial Agent; S5: Photographic Evidence from Laboratory Controls; S6: Statistical Evidence for Silver-Dependent Concentration–Response Relationship; S7: Summary Logic Chain; S8: Acknowledged Limitation and Future Controls; S9: References Cited in This Supplementary Document; S10: Images.
