# Supplementary material for: Green Synthesis of Silver Nanoparticles from Aloe vera: Antibacterial Potential Against Cyanobacteria from an Andean Lagoon
Source: Life (Basel). 2026 Jul 7;16(7):1132. doi: 10.3390/life16071132 (PMC13412621; doi:10.3390/life16071132)
Supplement: Supplementary file 1 [file life-16-01132-s001.zip › life-4190267-supplementary.pdf]

SUPPLEMENTARY MATERIAL

Evidence Supporting the Antibacterial Role of Silver Nanostructures and the Synthesis-Only Roles of Aloe vera Extract and Polyvinylpyrrolidone

Arnold Solano, Antonio Vega, José Davalos-Monteiro, Daniel Cabrera-Valle, Carlos Loyo-Dávila, Lenin Ramírez-Cando, Fernando Villalba-Meneses, Diego Almeida-Galárraga, Vladimir Bonilla, MarÍa Baldeon-Calisto, Raúl Dávalos Monteiro, Patricia Acosta-Vargas

Supporting Manuscript: Green Synthesis of Silver Nanoparticles from Aloe vera: Antibacterial Potential Against Cyanobacteria from an Andean Lagoon

S1. Purpose and Scope of This Supplementary Document

This supplementary document addresses a scientifically relevant question raised during peer review: whether the antibacterial inhibition of cyanobacteria observed in this study can be exclusively attributed to the silver nanostructures synthesized (AgNPs), rather than to the biological (*Aloe vera* extract) or polymeric (polyvinylpyrrolidone, PVP) reagents used in their preparation.

We present here four complementary lines of evidence: (1) photographic documentation of control versus treated cultures; (2) clarification of the distinct synthesis roles of each component; (3) literature-based rationale explaining why neither *Aloe vera* extract nor PVP can be considered the primary antibacterial agent under the conditions employed; and (4) statistical data from the concentration–response model supporting silver as the active species.

S2. Functional Roles of Each Component in the Synthesis System

The experimental system used in this study comprised four main components. It is critical to distinguish between those that serve as synthesis reagents and those that represent the active antibacterial species. Table S1 summarizes these roles.

Table S1. *Functional roles of each component in the synthesis and antibacterial assay.*

Component	Role in synthesis	Independent antibacterial role	Key reference(s)
Aloe vera extract (aqueous, 10% v/v, 25°C)	Reducing agent — polyphenols, aloin, and flavonoids donate electrons to reduce $Ag^+ \rightarrow Ag^0$	Not demonstrated under these conditions (aqueous extraction, dilute concentration)	Lawrence et al. (2009); Tippayawat et al. (2016)
PVP (10 kDa, 0.1% w/v)	Stabilizing agent — steric hindrance prevents AgNP agglomeration	None — chemically inert polymer; no biocidal activity without an active species (e.g., iodine)	Teodorescu & Bercea (2015); Al-Adham & Gilbert (1986)
$AgNO_3$ (6 mM) → AgNPs (γ solution, pH 8.45)	Silver precursor; reduced to silver nanostructures during synthesis	**Primary antibacterial agent** — oligodynamic effect via ROS, membrane disruption, ATP inhibition, Ag^+ ion release	Reidy et al. (2013); Prasher et al. (2018); Dong et al. (2019)
Z8 liquid medium	Cyanobacterial growth medium — provides $NaNO_3$, $Ca(NO_3)_2$, EDTA, trace elements	None	Temraleeva et al. (2016)

Note: *Aqueous Aloe vera extraction at 25°C with 10% v/v concentration produces substantially lower phenolic and anthraquinone yields than ethanol-based protocols. PVP exerts no biocidal function in the absence of an active antimicrobial species such as elemental iodine.*

S3. Why Aloe vera Extract Is Not the Antibacterial Agent

S3.1 Extraction conditions determine bioactivity

The antimicrobial activity of *Aloe vera* is highly dependent on extraction solvent and concentration. Lawrence et al. (2009) demonstrated that the ethanolic extract — not the aqueous extract — yields the greatest antibacterial activity, with inhibition zones of 23.00 mm against *Pseudomonas aeruginosa* and 12.66 mm against *Escherichia coli.* In contrast, the acetone extract showed limited or no activity against most tested organisms. The aqueous extract consistently showed intermediate or negligible results.

In our protocol, *Aloe vera* pulp was extracted in distilled water at 25°C for 35 minutes, producing an aqueous extract used at 10% v/v in the final reaction mixture. This represents a substantially diluted, solvent-poor extraction relative to the conditions required for independent bactericidal activity against photosynthetic microorganisms. Ahmad et al. (2025) reported an IC_{50} of 28.35 µg/mL for isolated aloe-emodin against *Staphylococcus epidermidis* — a concentration that exceeds the effective amount present in a 10% aqueous extract by several orders of magnitude when diluted further into 6.5 mL of assay volume.

S3.2 Role of aloin in silver reduction

The principal mechanism through which *Aloe vera* contributes to AgNP synthesis is the electron-donating capacity of aloin (a glucoside), aloe-emodin (a free anthraquinone), and polyphenolic compounds. These biomolecules reduce Ag^+ ions to Ag^0, simultaneously serving as surface-capping agents that stabilize nascent nanoparticle nuclei (Tippayawat et al., 2016; Iravani et al., 2014). This chemical transformation is the functional endpoint of *Aloe vera*'s role in the system. Once reduction is complete, the extract compounds remain adsorbed

on the nanoparticle surface as organic capping moieties, as confirmed by the detection of C, O, and N in our EDX spectrum.

Critically, the independent antimicrobial potential of these phytochemicals is not preserved in the same functional form after they participate in the reduction reaction: their reactive groups (hydroxyl, carbonyl) are oxidized during Ag^+ reduction, diminishing their independent bioactivity.

S4. Why Polyvinylpyrrolidone (PVP) Is Not the Antibacterial Agent

PVP is a chemically inert, non-ionic synthetic polymer consisting of repeating N-vinylpyrrolidone units. Its antimicrobial relevance exists exclusively in the context of PVP-iodine (povidone-iodine, PVP-I), where it serves as a carrier and controlled-release vehicle for elemental iodine — which is the actual biocidal species. The Al-Adham & Gilbert (1986) classic study demonstrated that antimicrobial activity in PVP solutions is a direct function of the free iodine concentration, not of PVP itself.

In our synthesis, **no iodine was used**. PVP at 0.1% w/v (10 kDa) functions solely as a steric stabilizer. Its pyrrolidone carbonyl groups coordinate to the AgNP surface, creating a polymeric shell that: (i) prevents van der Waals-driven agglomeration; (ii) maintains colloidal dispersion; and (iii) controls the kinetics of Ag^+ ion release from the nanoparticle surface. This last point is relevant: PVP's dense coating actually *reduces* the release rate of Ag^+ ions compared to citrate- or dextran-coated AgNPs (Ferreira et al., 2023), meaning that if anything, our PVP stabilization slightly attenuates — rather than amplifies — the silver-mediated antibacterial effect.

S5. Photographic Evidence from Laboratory Controls

The following descriptions correspond to the photographic record maintained throughout the two-month experimental period. Cyanobacterial biomass is directly observable via the green coloration of the culture medium, which arises from chlorophyll *a*, phycocyanin, and carotenoids. The intensity and saturation of this green color provides a reliable visual proxy for viable cell density, as confirmed by absorbance measurements at 630 nm.

S5.1 Control cultures — Z8 + cyanobacteria + Aloe vera extract + PVP (no AgNPs)

Bottles containing Z8 medium, cyanobacteria, *Aloe vera* aqueous extract (10% v/v), and PVP (0.1% w/v) — but no silver nitrate and therefore no AgNPs — maintained dense green coloration throughout the two-month observation period photographs. This is the critical visual control: it demonstrates that neither *Aloe vera* extract nor PVP, at the concentrations employed, inhibited cyanobacterial growth. Robust photosynthetic pigmentation and visible gas bubble formation (consistent with photosynthetic O_2 production) confirm continued metabolic activity.

S5.2 Low-dose AgNP treatment — partial inhibition (10 µL)

At the lowest AgNP dose (10 µL of γ solution, nominal silver concentration ≈ 0.27 mg/mL), cyanobacterial growth was partially inhibited but not eliminated. Cultures showed lighter green coloration relative to controls, and visible settling of green particulate matter to the bottom of the tube — consistent with cellular stress, loss of buoyancy regulation (gas vesicle disruption), and partial cell lysis. This single tube was the only sample in which bacterial growth was detected after the full incubation period.

S5.3 Complete inhibition at ≥20 µL AgNP dose

At volumes ≥20 µL (nominal MIC = 1.77 mg/mL), culture media appeared near-transparent, with no detectable green coloration. This visual outcome corresponds to the complete inhibition of cyanobacterial growth confirmed by absorbance at 630 nm and TDS measurements. The absence of green pigment indicates cessation of chlorophyll *a* synthesis and/or degradation of photosynthetic apparatus — outcomes consistent with AgNP-mediated oxidative stress and membrane disruption.

Figure S1. *Photographic documentation of experimental samples.*

Images are found in high quality in S10.

IMAGE PANEL A Control culture (Z8 + cyanobacteria + Aloe vera + PVP; NO AgNPs) Dense green coloration = active cyanobacterial growth Source: Laboratory photographs, April 2026	IMAGE PANEL B Low-dose AgNP (10 µL) Partial inhibition visible Lighter color, green precipitate settling Source: Laboratory photographs, April 2026	IMAGE PANEL C High-dose AgNP (≥20 µL) Complete inhibition Near-transparent medium Source: Laboratory photographs, April 2026

Note: *Photographs correspond to the experimental samples described in the main manuscript (Figure 6)*

S6. Statistical Evidence for Silver-Dependent Concentration–Response Relationship

The concentration–response data presented in the main manuscript provide additional evidence that the antibacterial effect is a function of silver nanostructure concentration rather than an indirect effect of *Aloe vera* or PVP, both of which were present at a fixed concentration across all experimental groups.

S6.1 Experimental design consideration

All 18 experimental samples contained identical amounts of Z8 medium (6 mL), cyanobacteria culture (0.5 mL), and — by construction of the AgNP solution — proportional amounts of PVP and residual *Aloe vera* extract carried over from the γ solution. The only variable systematically changed across samples was the volume of γ AgNP solution added (10 µL to 900 µL). If PVP or *Aloe vera* were responsible for inhibition, all samples would exhibit similar inhibitory effects regardless of volume — as both scale proportionally with the AgNP solution. Instead, a clear, statistically significant dose–response relationship was observed.

S6.2 Statistical model results

Table S2. *Linear regression results supporting silver concentration as the predictor of cyanobacterial inhibition.*

Variable	Mean estimate	Std. error	t-value	P-value
Intercept (a)	1.81206	6.01	29.419	**<0.001**
1/[AgNPs] (b)	−17.15956	0.0093	−10.509	**<0.001**
Effect size (R^2)	**0.87** ($p = 1.923 \times 10^{-8}$)			
F-ratio	**105.7 on 1 and 16 df**			

Note: *Model: log(Abs630nm) = a + b/[AgNPs]. The model fits an exponential decay relationship between AgNP concentration and optical density. Statistical analysis performed in RStudio (v2023.09.1+494). PVP and Aloe vera extract concentrations were constant across all samples and therefore cannot explain the observed dose-dependent variation.*

The exponential decay model (effect size $R^2 = 0.87$, $F = 105.7$, $p < 0.001$) demonstrates that 87% of the variance in cyanobacterial absorbance is explained by silver nanoparticle concentration alone. This is a statistically robust relationship incompatible with a constant background effect from fixed-concentration reagents (PVP, *Aloe vera*). Complete inhibition at ≥20 µL (nominal MIC = 1.77 mg/mL) is consistent with the oligodynamic threshold concentrations reported for AgNPs in the literature.

S7. Summary Logic Chain

The following five points constitute the logical chain supporting silver as the active antibacterial agent:

1. **Control cultures confirm PVP and Aloe vera do not inhibit growth.** Bottles containing Z8 medium, cyanobacteria, aqueous *Aloe vera* extract, and PVP — but no $AgNO_3$ — remained visually green with continued cyanobacterial viability throughout the two-month experiment.
2. **Inhibition follows a silver dose–response relationship.** Only AgNP concentration varies meaningfully across experimental groups; PVP and *Aloe vera* scale proportionally in all samples. The statistically significant dose–response ($R^2 = 0.87$, $p < 0.001$) is consistent only with silver as the independent variable.
3. **Extraction conditions preclude Aloe vera independent bactericidal activity.** Aqueous extraction at 25°C yields substantially lower concentrations of anthraquinones and phenolics compared to ethanol-based protocols. At 10% v/v further diluted in 6.5 mL assay volume, the effective phenolic concentration is well below documented minimum inhibitory concentrations for photosynthetic microorganisms.
4. **PVP has no biocidal mechanism without an active species.** The antimicrobial literature consistently attributes PVP-mediated killing to the PVP-iodine complex, not to PVP itself. Without iodine, PVP is chemically inert toward bacteria. Furthermore, PVP coating of AgNPs is known to attenuate rather than enhance Ag^+ ion release.
5. **UV-Vis and EDX confirm successful silver nanostructure synthesis.** SPR peaks between 425–460 nm and EDX silver content of 59.96 wt% confirm the presence of silver-containing nanostructures as the distinguishing feature of AgNP-treated vs. control samples.

S8. Acknowledged Limitation and Future Controls

We fully acknowledge, as stated in Section 3.6 of the main manuscript, that the absence of individual component controls — i.e., groups receiving $AgNO_3$ alone, PVP alone, and aqueous *Aloe vera* extract alone — is a limitation of the present exploratory study. While the evidence presented in this supplementary document strongly supports silver as the active agent, definitive mechanistic attribution requires the inclusion of these negative controls.

These controls will be incorporated into a follow-up study currently in preparation. The planned experimental design will include: (i) cyanobacteria + $AgNO_3$ solution (no reduction/synthesis), (ii) cyanobacteria + aqueous *Aloe vera* extract at equivalent concentrations, (iii) cyanobacteria + PVP solution at equivalent concentrations, (iv) fully synthesized AgNPs (current study conditions), and (v) untreated cyanobacteria control. This expanded design will allow statistical discrimination between the contributions of each component.

S9. References Cited in This Supplementary Document

Al-Adham, I.S.I. & Gilbert, P. (1986). Effect of polyvinylpyrrolidone molecular weight upon the antimicrobial activity of povidone-iodine antiseptics. *International Journal of Pharmaceutics,* 34(1–2), 45–49.

Ahmad, M.M. et al. (2025). Quantification of aloe-emodin in aloe-vera extract and antimicrobial activities studies. *Research Journal of Pharmacy and Technology,* 165–172.

Dong, Y. et al. (2019). Antibacterial activity of silver nanoparticles of different particle size against *Vibrio natriegens.* PLOS ONE, 14, e0222322.

Ferreira, A.M. et al. (2023). How similar is the antibacterial activity of silver nanoparticles coated with different capping agents? *RSC Advances,* 13(16), 10542–10555.

Iravani, S. et al. (2014). Synthesis of silver nanoparticles: Chemical, physical and biological methods. *Research in Pharmaceutical Sciences,* 9, 385.

Lawrence, R. et al. (2009). Isolation, purification and evaluation of antibacterial agents from aloe vera. *Brazilian Journal of Microbiology,* 40, 906–915.

Liao, C. et al. (2019). Bactericidal and cytotoxic properties of silver nanoparticles. *International Journal of Molecular Sciences,* 20(2), 449.

Prasher, P. et al. (2018). Oligodynamic effect of silver nanoparticles: A review. *BioNanoScience,* 8, 951–962.

Reidy, B. et al. (2013). Mechanisms of silver nanoparticle release, transformation and toxicity. *Materials,* 6, 2295–2350.

Teodorescu, M. & Bercea, M. (2015). Poly(vinylpyrrolidone) — a versatile polymer for biomedical and beyond medical applications. *Polymer-Plastics Technology and Engineering,* 54(9), 923–943.

Temraleeva, A. et al. (2016). Modern methods for isolation, purification, and cultivation of soil cyanobacteria. *Microbiology,* 85, 389–399.

Tippayawat, P. et al. (2016). Green synthesis of silver nanoparticles in Aloe vera plant extract and their synergistic antibacterial activity. *PeerJ,* 4, e2589.

S10. Images

IMAGES PANEL A Control culture (Z8 + cyanobacteria + Aloe vera + PVP; NO AgNPs) Dense green coloration = active cyanobacterial growth Source: Laboratory photographs, April 2026

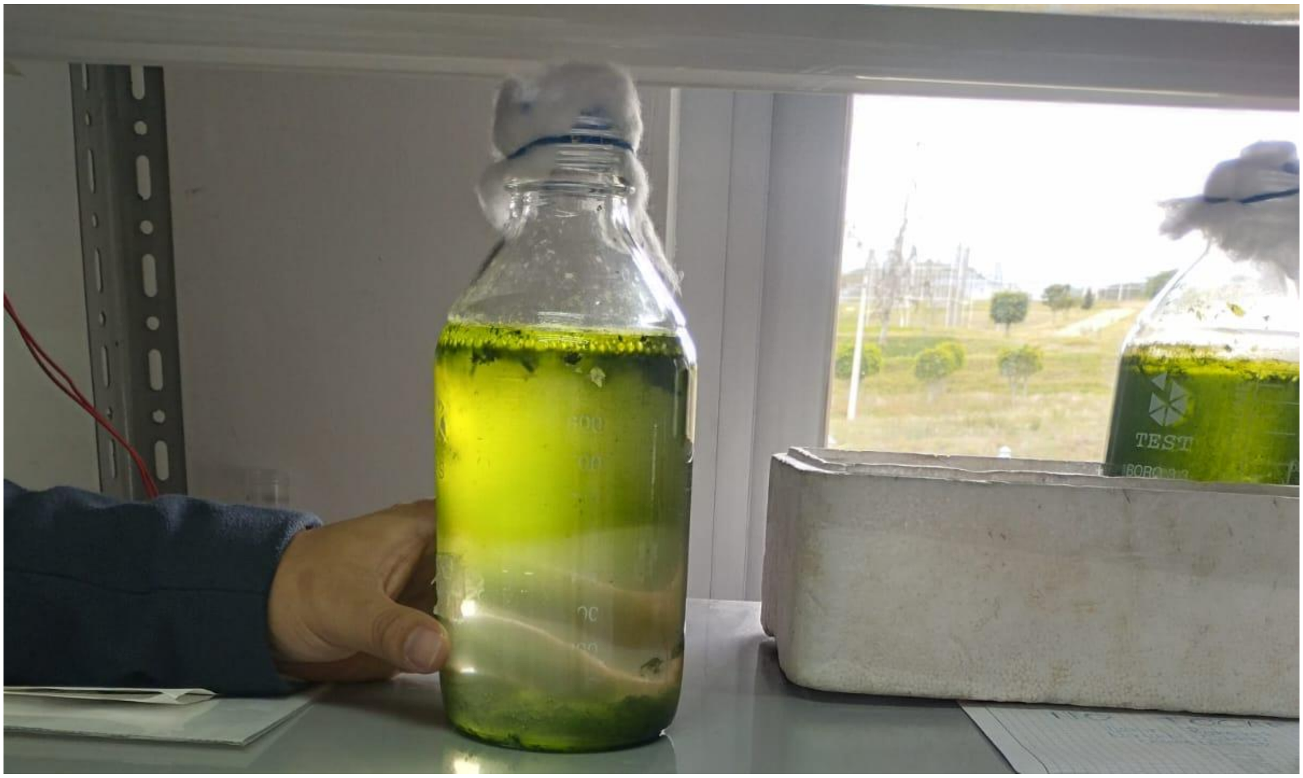
TEST

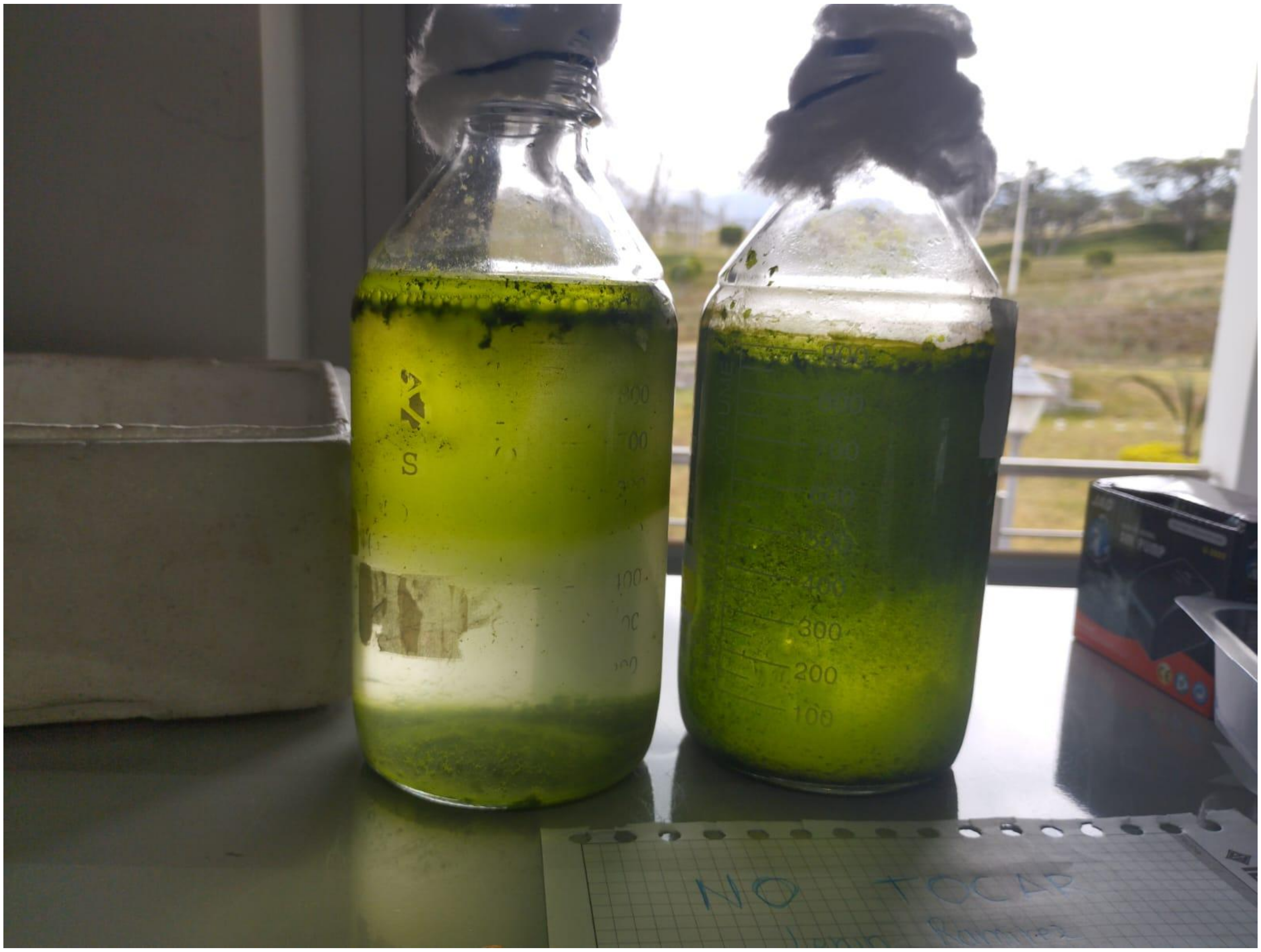
NO TOCAR

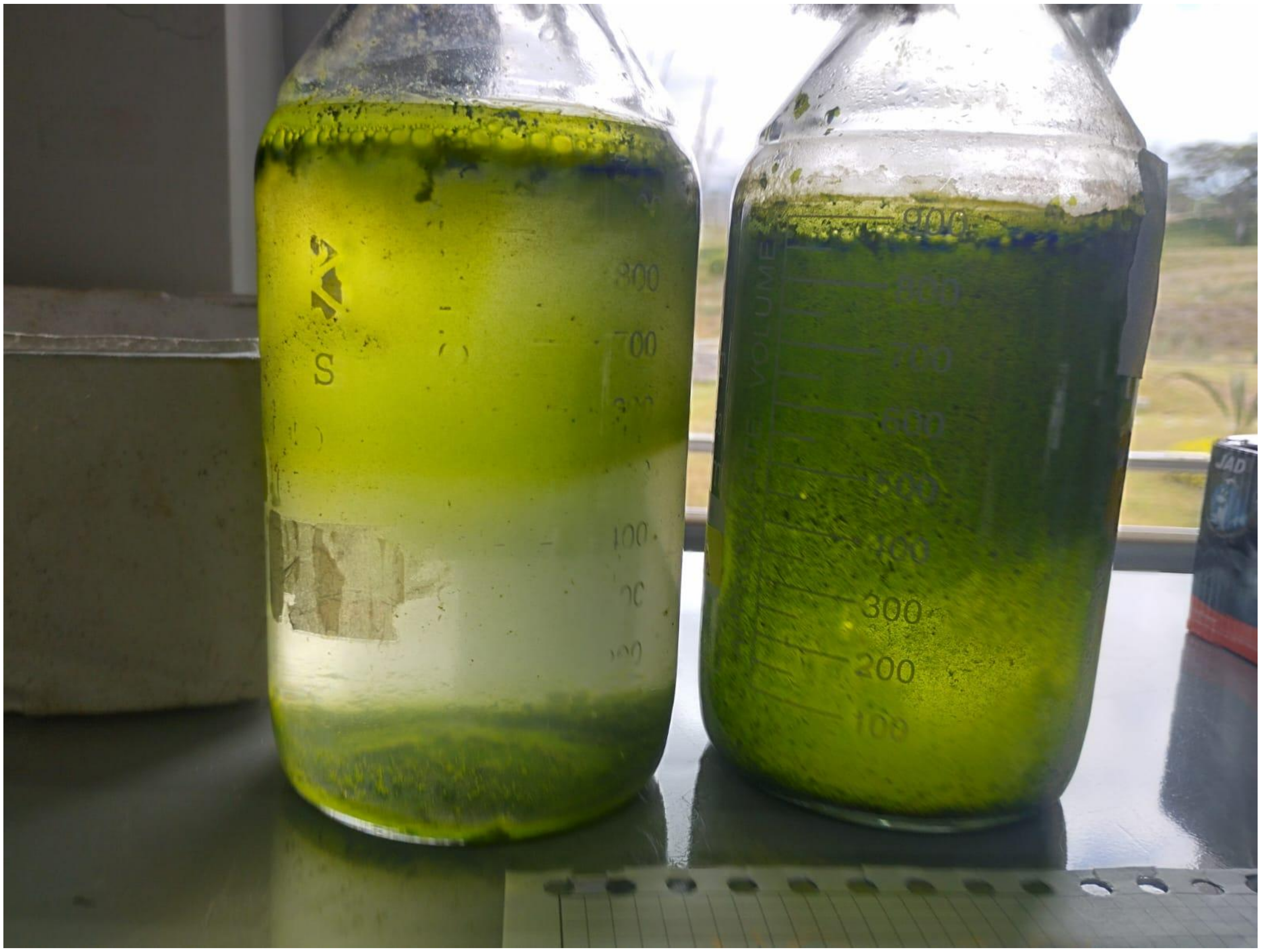
800
700
300
200

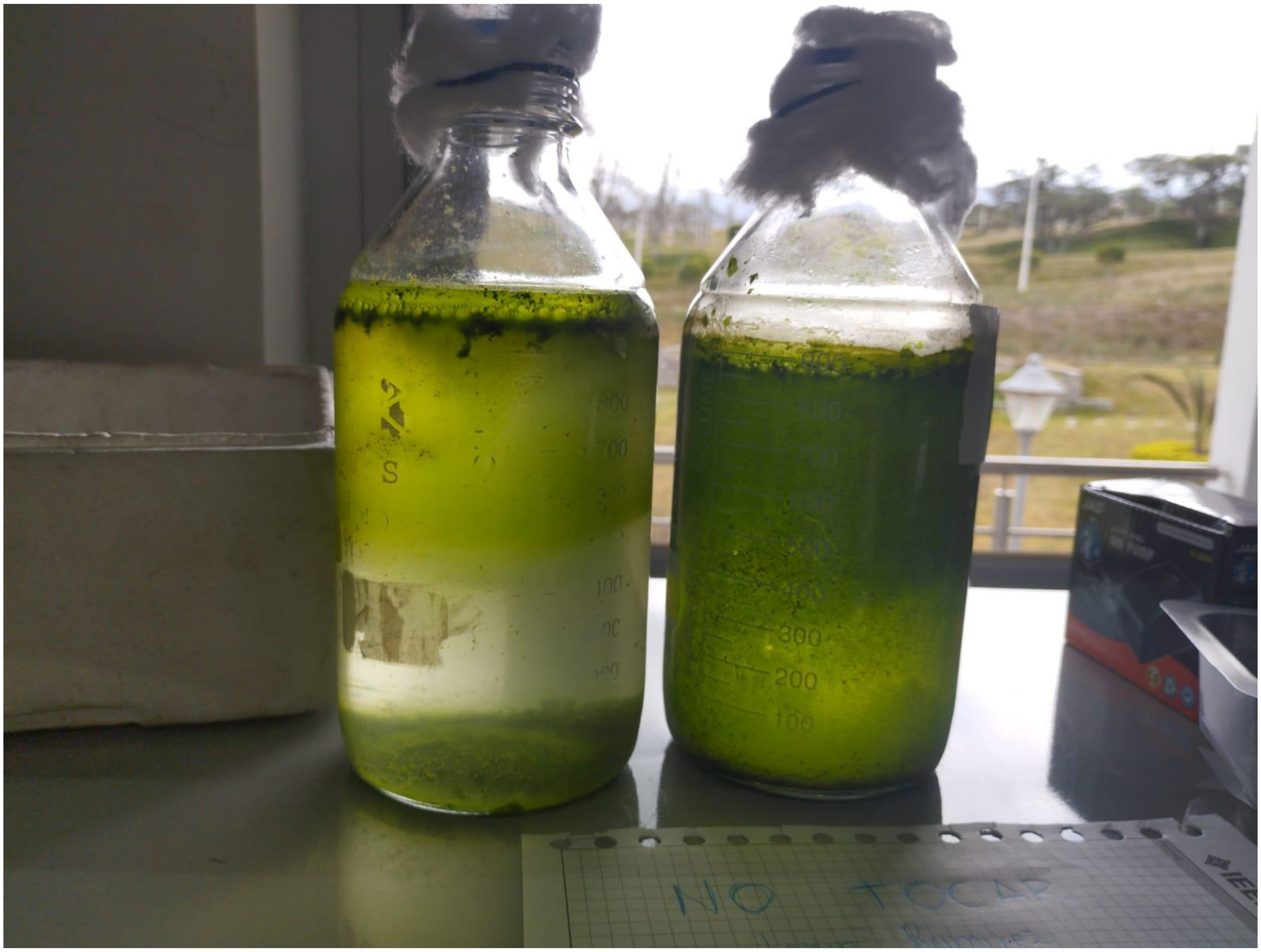
NO TOCAR

IMAGE PANEL C High-dose AgNP (≥20 µL) Complete inhibition Near-transparent medium Source: Laboratory photographs, April 2026

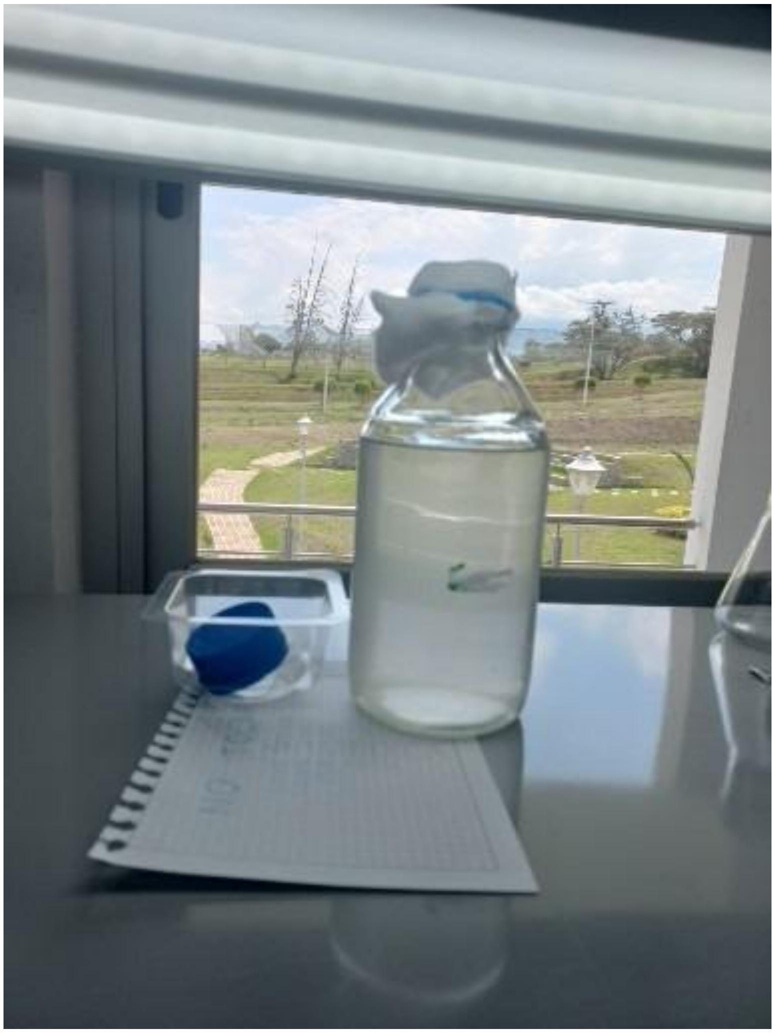

IMAGE PANEL B Low-dose AgNP (10 µL) Partial inhibition visible Lighter color, green precipitate settling Source: Laboratory photographs, April 2026

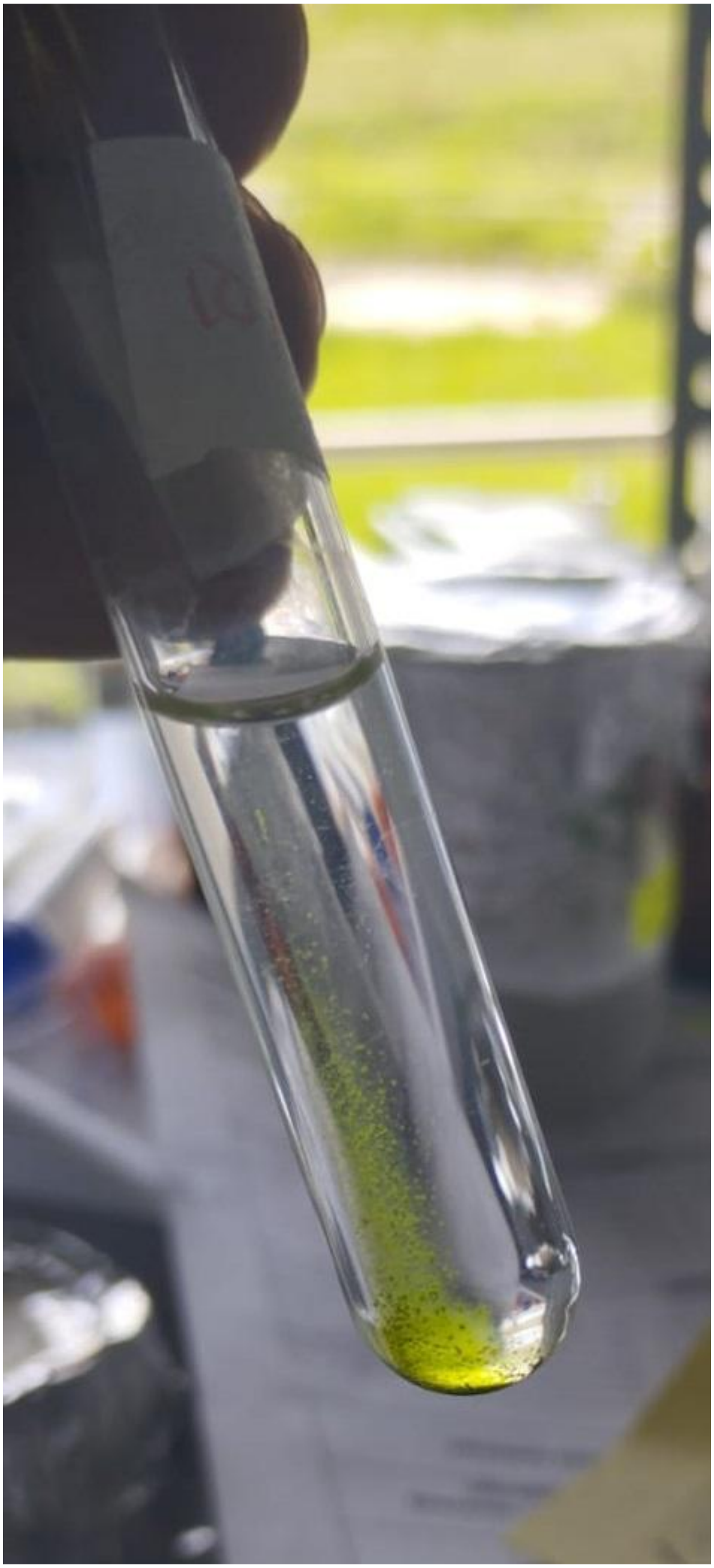

Solano et al. (2026). Data available at:
